# Epstein-barr virus infected gastric adenocarcinoma expresses latent and lytic viral transcripts and has a distinct human gene expression profile

**DOI:** 10.1186/1750-9378-7-21

**Published:** 2012-08-28

**Authors:** Weihua Tang, Douglas R Morgan, Michael O Meyers, Ricardo L Dominguez, Enrique Martinez, Kennichi Kakudo, Pei Fen Kuan, Natalie Banet, Hind Muallem, Kimberly Woodward, Olga Speck, Margaret L Gulley

**Affiliations:** 1 Department of Pathology & Laboratory Medicine, University of North Carolina, 913 Brinkhous-Bullitt Building, Chapel Hill, NC, 27599-7525, USA; 2 Gastroenterology and Hepatology Division, Department of Medicine, University of North Carolina, Chapel Hill, NC, 27599-7080, USA; 3 Surgical Oncology, Lineberger Comprehensive Cancer Center, P1150 Physician’s Office Bldg. 170 Manning Dr, Chapel Hill, NC, 27599-7213, USA; 4 Department of Gastroenterology, Western Regional Hospital, Santa Rosa de Copan, Honduras; 5 Department of Gastroenterology, Hospital Evangelico, Apartado Postal # 15, Siguatepeque, Comayagua, Honduras; 6 Department of Pathology, Kobe-Tokiwa University, Nagata-ku, Kobe, Hyōgo, Japan; 7 Biostatistics, Lineberger Comprehensive Cancer Center, University of North Carolina, Chapel Hill, NC, 27599-7295, USA; 8 Department of Pathology & Laboratory Medicine, Lineberger Comprehensive Cancer Center, University of North Carolina, 913 Brinkhous-Bullitt Building, Chapel Hill, NC, 27599-7525, USA

**Keywords:** Gastric adenocarcinoma, Epstein-barr virus, RNA expression profile, Stromal cells, Pharmacogenetic test

## Abstract

**Background:**

EBV DNA is found within the malignant cells of 10% of gastric cancers. Modern molecular technology facilitates identification of virus-related biochemical effects that could assist in early diagnosis and disease management.

**Methods:**

In this study, RNA expression profiling was performed on 326 macrodissected paraffin-embedded tissues including 204 cancers and, when available, adjacent non-malignant mucosa. Nanostring nCounter probes targeted 96 RNAs (20 viral, 73 human, and 3 spiked RNAs).

**Results:**

In 182 tissues with adequate housekeeper RNAs, distinct profiles were found in infected *versus* uninfected cancers, and in malignant *versus* adjacent benign mucosa. EBV-infected gastric cancers expressed nearly all of the 18 latent and lytic EBV RNAs in the test panel. Levels of *EBER1* and *EBER2* RNA were highest and were proportional to the quantity of EBV genomes as measured by Q-PCR. Among protein coding EBV RNAs, *EBNA1* from the Q promoter and *BRLF1* were highly expressed while *EBNA2* levels were low positive in only 6/14 infected cancers. Concomitant upregulation of cellular factors implies that virus is not an innocent bystander but rather is linked to NFKB signaling (*FCER2, TRAF1*) and immune response *(TNFSF9, CXCL11, IFITM1, FCRL3, MS4A1 and PLUNC)*, with *PPARG* expression implicating altered cellular metabolism. Compared to adjacent non-malignant mucosa, gastric cancers consistently expressed *INHBA, SPP1, THY1, SERPINH1, CXCL1, FSCN1, PTGS2 (COX2), BBC3, ICAM1, TNFSF9, SULF1, SLC2A1, TYMS*, three collagens, the cell proliferation markers *MYC* and *PCNA*, and EBV *BLLF1* while they lacked *CDH1 (E-cadherin), CLDN18*, *PTEN, SDC1* (CD138), *GAST* (gastrin) and its downstream effector *CHGA* (chromogranin). Compared to lymphoepithelioma-like carcinoma of the uterine cervix, gastric cancers expressed *CLDN18, EPCAM, REG4, BBC3, OLFM4, PPARG*, and *CDH17* while they had diminished levels of *IFITM1* and *HIF1A*. The druggable targets ERBB2 (Her2), MET, and the HIF pathway, as well as several other potential pharmacogenetic indicators (including EBV infection itself, as well as *SPARC, TYMS, FCGR2B* and *REG4*) were identified in some tumor specimens.

**Conclusion:**

This study shows how modern molecular technology applied to archival fixed tissues yields novel insights into viral oncogenesis that could be useful in managing affected patients.

## Background

Gastric cancer is the second leading cause of global cancer mortality with nearly one million new cases per year [1,2]. Approximately ten percent of gastric adenocarcinomas are Epstein-Barr virus (EBV) infected, and EBV is considered a class 1 oncogenic pathogen by the World Health Organization [3-6]. Incidence is rising for those cancers in the proximal segment of the stomach (cardia, corpus) where EBV is more frequently involved [7-14].

Recent data from the National Cancer Institute’s cancer surveillance program shows a worrisome rise in gastric cancer incidence among young adults in the US [7,8,15]. Emerging targeted therapy makes it all the more important to identify infected cancers and to characterize biochemical defects such as ERBB2 overexpression that increases likelihood of response to trastuzumab in metastatic gastric cancer patients [16-18]. EBV-infected compared to uninfected gastric cancer has a favorable prognosis [19], and clinical trials are beginning to explore virus-targeted therapy such as 1) infused EBV-specific cytotoxic T cells or NK cells [20-23], 2) reversing the EBV-related methylator phenotype [24], 3) triggering lytic viral replication that could then incite the body’s innate and adaptive immune responses to kill infected tumor cells [25-33], and 4) lytic induction therapy co-administered with antiviral nucleoside analog such as gancyclovir that is phosphorylated and thus activated by viral kinases promoting cytotoxicity [34-41].

Clinical trials examining the efficacy of targeted therapy would benefit from laboratory assays that help identify candidates likely to respond, and could benefit from laboratory assays that signify the effect of intervention on the intended biochemical pathways. Modern molecular technology now permits clinical-grade analysis of multiple pertinent analytes via RNA expression profiling [42]. Device manufacturers have produced sensitive, specific and customizable probe arrays to simultaneously measure multiple RNAs, including non-coding RNAs like EBV-encoded RNA 1 or 2 that are abundantly expressed in infected tumors. Recent progress in quality assurance strategies have matured to the point that RNA expression profiles are being implemented in clinical laboratory settings [42].

To be practical in clinical settings, an assay must be applicable to routinely collected specimens such as archival, paraffin-embedded tissue [42]. In the current study, we measured viral and human gene expression in archival gastric cancers and in adjacent mucosa and controls to develop a test systems that might be used to reliably characterize signatures predictive of response to targeted therapy. A 96-RNA array test system that we dub the Gastrogenus v1™ panel was customized to measure pertinent latent and lytic viral RNAs alongside clinically relevant human mRNAs that were previously reported to be 1) gastric cancer specific, 2) indicative of inflammation, and/or 3) predictive of response to specific medications. These assays, as well as spiked and endogenous control RNAs, were measured in macrodissected paraffin sections using the Nanostring nCounter test system [43-45]. Correlative histologic and molecular studies were done to demonstrate that the test system performed as expected. Our findings show that EBV-related cancers express more latent and lytic transcripts than were previously recognized, and that infected cancers have unique biologic characteristics compared with uninfected cancers. Two major subtypes of cancer were found, implying that gastric cancer early detection strategies or monitoring tests could be tailored to detect the pertinent signatures characterizing major molecular subtypes. Finally, pilot data reveals expression of selected viral and cancer-related genes in adjacent non-malignant mucosa, suggesting a field effect that could be important in cancer development or maintenance.

## Results

**Figure 1:**
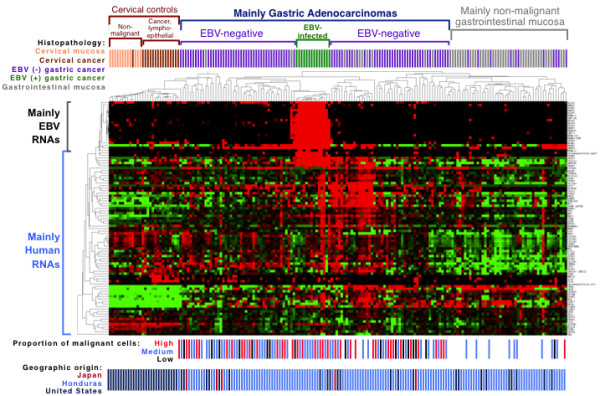
** Expression profiles of 182 tissues for 20 viral genes and 73 human genes.** A heat map displays unsupervised hierarchical clustering of each tissue in a separate column, and each RNA in a separate row. The data is median-centered with red indicating relative overexpression and green indicating relative under-expression for each gene. Correlative data above the map indicates histopathologic classification with further subclassification of the gastric cancer cohort into 14 EBV infected and 104 EBV negative cancers based on EBV DNA levels. Below the map, each gastric cancer is categorized by the proportion of malignant cells, and geographic origin of each tissue is shown.

Gene expression profiling was performed on a total of 326 tissues including 187 gastric cancers, 17 lymphoepithelioma-like cervical cancers, and 118 matched non-malignant mucosa from the same surgical procedure (when available). After data normalization, a heat map of the 182 tissues having the best quality RNA, as judged by highest average level of four housekeeping RNAs, revealed patterns of gene expression that differed in gastric *versus* cervical control tissues. Furthermore, in both the gastric and cervical clusters, malignant and non-malignant tissues tended to cluster together, supporting the ability of the nCounter test system to measure clinically important biologic features. (See Figure 1.)

**Figure 2:**
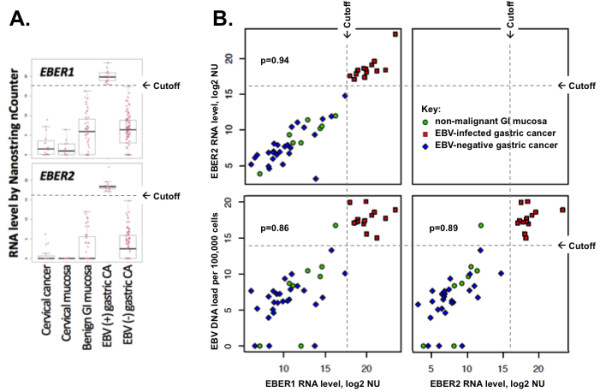
** EBV-encoded RNA levels are high in infected gastric cancer and are proportion to EBV genome level. A**. Box plots of *EBER1* and *EBER2* in benign and malignant tissues reveal that EBV-infected gastric cancer has substantially higher levels of *EBER1* and *EBER2* non-coding RNAs than do uninfected cancers and control tissues. Proposed thresholds for *EBER1* or *EBER2* are shown beyond which a gastric cancer could reliably be designated as EBV-infected. Each dot represents an individual analytic result on a log2 normalized unit (NU) scale. **B**. Pairwise comparison of *EBER1* and *EBER2* RNA levels by Nanostring nCounter array and EBV DNA viral load by Q-PCR reveals a linear association between levels of each of these analytes. Pearson correlation coefficients (P >0.86) are shown. The previously validated level of EBV DNA viral load is shown beyond which *EBER* was always localized to malignant cells by *EBER in situ* hybridization (threshold of 10,558 EBV genomes per 100,000 cells, which is equivalent to 13.37 on this log2 scale) [46]. Proposed cutoffs for RNA levels are indicated for both *EBER1* (200,000 NU, or 17.61 on this log 2 scale) and *EBER2* (100,000 NU, or 16.61 on this log2 scale). The one outlier is a non-malignant gastric mucosa that was located adjacent to an EBV-infected gastric cancer, and this mucosa had an EBV DNA load equivalent to that of infected cancers, but it would have been correctly excluded from the EBV-infected cancer group if either *EBER1* or *EBER2* RNA levels were used, or if histology were used, to screen for EBV-related malignancy.

One group of gastric carcinomas overexpressed virtually all of the EBV RNAs. To determine which gastric cancers should be designated as EBV-infected, the 71 tissues with the highest combined levels of *EBER1* and *EBER2* RNA by Nanostring nCounter array were further examined for EBV genome levels within the same tissue by Q-PCR. There was a linear relationship between the amount of *EBER1* and *EBER2* RNA and the amount of EBV genome. (See Figure 2.) Our previously established cutoff [46] for the level of EBV genome corresponding to localization of virus to malignant cells resulted in 14 cancers being placed in the EBV-infected category. The remaining gastric cancers were called EBV-negative, and among them the highest recorded RNA levels were 174,016 for *EBER1* and 27,972 for *EBER2*. In contrast, among the EBV-infected gastric cancers the lowest *EBER1* level was 263,589 and the lowest *EBER2* level was 140,081. Proposed cutoffs for identifying a tissue as EBV-infected are shown in Figure 2. 

### Genes overexpressed in EBV-infected versus EBV-negative gastric cancer

Twenty eight genes were significantly differentially expressed in EBV-infected cancers compared to the EBV negative gastric cancers (p < 0.05). Interestingly, all 28 were upregulated rather than downregulated in the infected cancers, and this bias is explained at least in part by our selection of positive rather than negative markers of infection when choosing the RNAs to be profiled for this study. Failure to identify any downregulated genes was still surprising given reports that EBV is associated with a CpG island methylator phenotype and additionally the virus can destabilize cellular mRNAs globally [47].

**Figure 3:**
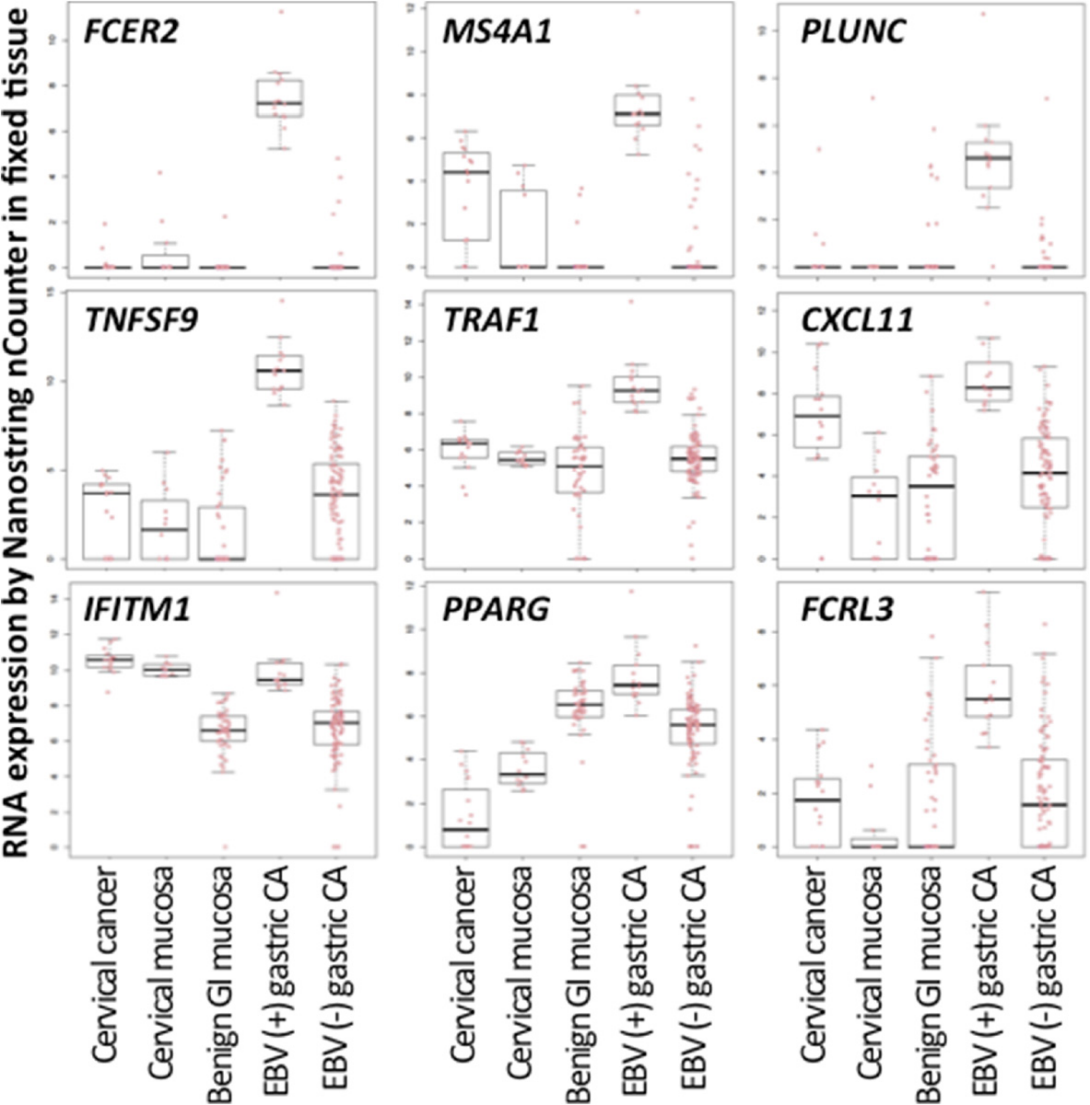
** Multiple human RNAs are over-expressed in EBV-infected gastric cancer compared to EBV-negative cancer.** Box plots demonstrate the human RNAs levels in infected compared to uninfected gastric cancers and controls that include lymphoepithelioma-like cervical cancer, cervical mucosa, and benign gastrointestinal mucosa. Each dot represents an individual analytic result on a log2 normalized unit scale.

Among the genes significantly upregulated in infected cancers were all 18 of the EBV RNAs tested, as well as cytomegalovirus pp65 (UL83). The cytomegalovirus pp65 (UL83) result is likely to be false positive (suspected to be probe cross hybridization), as evidenced by absence of another lytic RNA, cytomegalovirus pol (UL54), in the EBV-infected cancers. Furthermore, UL83 but not UL54 was expressed in EBV infected but not in EBV-negative cell line controls (data not shown). Another possible explanation for false positive viral RNA expression is probe crossreactivity with viral DNA. Nine human RNAs were significantly upregulated in EBV-infected compared to EBV negative gastric cancers: *FCER2, MS4A1 (CD20), PLUNC, TNFSF9, TRAF1, CXCL11, IFITM1, PPARG*, and *FCRL3*. (See Figure 3).

### Genes differentially expressed in gastric cancer compared to non-malignant gastrointestinal mucosa

Twenty six genes were significantly dysregulated in gastric cancer compared to non-malignant gastric mucosa (p < 0.05). The human RNAs upregulated in gastric cancer were *INHBA, SPP1, THY1, SERPINH1, CXCL1, FSCN1, COL1A1, SPARC, COL1A2, PTGS2 (COX2), BBC3, ICAM1, TNFSF9, MYC, SULF1, SLC2A1, COL3A1, PCNA, and TYMS*, while the downregulated RNAs were *CDH1 (E-cadherin), CLDN18*, *CHGA* (chromogranin), *PTEN, SDC1* (CD138) and *GAST* (gastrin). The only viral factor that was differentially expressed was *BLLF1* which was significantly higher in cancer than in non-malignant gastric mucosa (p = 0.004). *BLLF1* encodes the late viral envelope protein gp350/220, suggesting that virions are significantly more prevalent in cancer than in non-malignant gastric tissue. *BLLF1* was not specific for gastric cancer, however, as it was also expressed in some benign and malignant cervical tissues, as well.

### Genes associated with gastric cancer compared to lymphoepithelioma-like cervical cancer

Nine genes were significantly dysregulated in gastric cancer compared to lymphoepithelioma-like cervical cancer (p < 0.05). The seven RNAs upregulated in gastric cancer were *CLDN18, EPCAM, REG4, BBC3, OLFM4, PPARG*, and *CDH17*, while the two downregulated genes were *IFITM1* and *HIF1A*.

### Patterns of latent and lytic viral gene expression in EBV infected gastric cancers

The 14 EBV-infected gastric cancers in this study consistently coexpressed virtually all of the EBV latent and lytic genes, which is somewhat surprising given that prior literature describes a somewhat restricted latency pattern [48-51]. It is feasible that the Nanostring nCounter analytic technology is more sensitive than traditional methods of detection.

**Figure 4:**
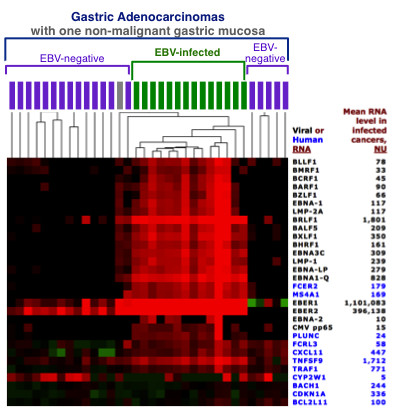
** Latent and lytic EBV genes are co-expressed in gastric cancer.** A portion of the heat map from Figure 1 is displayed in high contrast to decipher relative expression levels of EBV genes in the 14 EBV-infected gastric cancers and surrounding specimens. All tissues are gastric cancers except a single non-malignant gastric mucosa, shown in grey, dissected from the same paraffin block as an EBV-infected gastric cancer. Mean expression level of each RNA in the EBV-infected gastric cancer cohort is shown to the right of each gene symbol.

The most highly expressed viral RNA was *EBER1* at an average of over 1 million normalized units per EBV-infected cancer tissue, followed by *EBER2*, *BRLF1* and *EBNA1* from of the Q promoter. *EBNA2* was the least expressed viral RNA with a mean expression of only 10 normalized units per infected tissue and *EBNA2* was completely absent in 8 of the 14 infected gastric cancers. Patterns of viral gene expression are depicted in Figure 4.

### Geographic origin and tumor cell proportion are not preferentially associated with EBV status of gastric cancer

Below the heat map in Figure 1 is the distribution of gastric cancer cases by geographic origin from Honduras (n = 86), Japan (n = 5), or the United States (n = 17). There was no significant association between geographic origin and EBV-positive *versus* negative clustering of gastric cancers (Fisher’s exact test p = 0.9), suggesting that geographic origin is not the major driver of hierarchical clustering.

The bottom of Figure 1 also shows the distribution of EBV-infected *versus* EBV-negative gastric cancers classified by the proportion of malignant cells input into the expression profiling assay. There was no significant association between the proportion of malignant cells and the EBV-infected *versus* EBV-negative groups of gastric cancer. Surprisingly, the cancer tissues with low malignant cell content did not preferentially cluster with the non-malignant gastric tissues. Cancers with low malignant cell content (1 to 25% malignant cells) were distributed across various segments of the heat map along with cancers with medium (26 to 50%) or high (>50%) malignant cell content (Fisher’s exact test p = 0.5), suggesting that overall transcriptome features outweigh tumor cell proportion as the driver of hierarchical clustering.

Keeping in mind that the lymphoepithelioma-like cervical cancers in this study were rich in lymphoid stroma, as are many EBV-infected gastric cancers, it is remarkable that these two classes of cancer clustered separately from each other and also achieved reasonably good separation from adjacent non-malignant mucosa. For most genes in the panel, there is considerable overlap in levels across disease types. While profiles are more informative and more convincing than are individual transcript results, there is some overlap in profiles as well, signifying that profiling assay results must be correlated with histologic features in order to accurately classify a tissue as benign or malignant.

### Pharmacogenetic predictors and druggable targets

**Figure 5:**
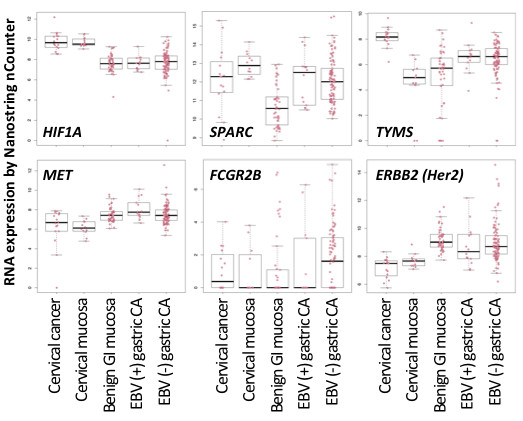
** Some gastric cancers have significant dysregulation of factors that show promise as pharmacogenetic predictors.** Box plots demonstrate expression of selected pharmacogenetic targets in infected versus non-infected gastric cancers as well as non-malignant gastric mucosa and cervical histopathologies. Each dot represents an individual analytic result on a log2 normalized unit scale.

EBV infection itself is considered an actionable target, at least for the 14/108 (13%) infected gastric cancers we identified. This study demonstrates a novel way to identify virus-infected cancers by RNA profiling of paraffin sections so that prognostic and predictive information may be considered in patient management decisions. Cellular factors of pharmacogenetic potential include the HIF pathway, *SPARC, TYMS, FCGR2B, MET,* and *ERBB2 (Her2)*. (See Figure 5). Compared with gastric cancers, cervical cancers tend to have higher levels of *HIF1A* indicating hypoxia response, although equally high levels in non-malignant cervical mucosa raise the possibility of *ex vivo* stimulation of this oxygen-sensing factor. Further study is needed to distinguish technical factors from *in vivo* upregulation that would warrant consideration of angiogenesis inhibitors.

We confirmed that *SPARC* is upregulated in gastric cancer compared to benign gastric mucosa. Response to docetaxel, a taxane drug that inhibits mitotic spindle assembly, is reportedly impacted by the amount of SPARC protein expression in gastric cancer [52]. Gastric and cervical cancers both had higher *thymydylate synthase (TYMS)* than did their respective benign mucosal counterparts. High *TYMS* levels reportedly contributes to acquired resistance to 5FU combination therapy [53].

A few gastric cancers had extremely high levels of the Fc receptor, *FCGR2B*, which could affect drug internalization and pharmacodynamics of therapeutic antibodies such as cetuximab *in vivo.* Four gastric cancers strongly expressed *MET,* and an additional eight cases strongly overexpressed expressed *ERBB2 (Her2)*, raising the possibility that this assay could predict response to tyrosine kinase inhibitor therapy.

## Discussion

This study used modern molecular methods to examine a large panel human and viral RNAs in gastric cancer. To our knowledge, this is the largest panel of viral gene products to be examined in concert with human RNAs in archival, paraffin embedded tissues. The EBV-infected subtype of gastric cancer is dramatically evident in the corresponding heat map created by unsupervised clustering, and EBV infection was confirmed by high EBV DNA viral loads in these tissues. Expression of selected viral and human genes in the cancers confirmed several known virus- and cancer-related effects and also revealed novel findings that shed light on pathogenesis and possible disease management strategies.

Surprisingly, the infected gastric cancers overexpressed all 18 of the latent and lytic EBV genes that were tested. We discovered high levels of *BRLF1* RNA (encoding the immediate early viral protein triggering lytic replication in concert with BZLF1) and moderately high levels of *BXLF1* (the viral thymidine kinase that converts penicyclovir to a toxic form, suggesting a mechanism for therapy) [54]. *BLLF1* (encoding the late viral envelope protein gp350/220) was expressed at moderate levels that were nevertheless significantly higher than in non-malignant mucosa, suggesting that EBV lytic infection is not abortive but rather is capable of producing the late viral envelope protein gp350/220. Among the latent genes, *EBNA1* from the Q promoter, *EBNA-LP, and EBNA3C* transcripts were most prevalent. *EBNA2* was focally detected at low level but was still significantly higher in infected than in uninfected gastric cancers. Prior histochemical work has generally not revealed protein-level expression of the EBNAs or lytic viral gene products, so further work is required to learn if these virally encoding RNAs are localized to malignant cells, lymphocytes, or possibly even to exosomes or virions in the extracellular milieu.

Compared to uninfected cancers, the infected cancers had significant upregulation of nine cellular factors (*FCER2, MS4A1 (CD20), PLUNC, TNFSF9, TRAF1, CXCL11, IFITM1, PPARG*, and *FCRL3*), implying that EBV is not an innocent bystander with respect to biochemical impact. The virus-associated changes we found were in pathways known to viral oncologists, namely NFKB and NOTCH signaling (*FCER2, TRAF1, PPARG*) and mucosal immune response (*PLUNC, TNFSF9, CXCL11, IFITM1, FCRL3*). MS4A1 (CD20) is B cell specific, reminding us that some of the factors upregulated in EBV-infected compared to uninfected gastric cancers could derive from stromal elements rather than from malignant epithelial cells. *PLUNC* was previously described as a tumor marker for gastric and nasopharyngeal carcinomas, and it encodes a secreted protein involved in innate immune response [55-57]. TNFSF9, a cytokine of the tumor necrosis factor family, stimulates T cell activation and triggers IFNG production which in turn induces the proinflammatory chemokine CXCL11 and the innate antiviral factor IFITM1. PPARG is as a nuclear receptor controlling glucose metabolism and microtubule networks, and it is a promising target for inhibitory drugs [58]. The *FCRL3* immune response gene is mutated in autoimmune diseases such as rheumatoid arthritis, lupus, and Grave’s disease.

Our findings support the work of Lee *et al* who found distinct human expression patterns in infected *versus* uninfected gastric cancers [10]. Although their study targeted protein and ours targeted RNA, our findings agreed with theirs for 4 of the 5 factors in common between the two studies (*BCL2, PTEN, CDH1, PTGS2*). There was a potential discrepancy for *ERBB2* that was significantly less frequently expressed in infected compared to uninfected gastric cancers when tested at the protein level [10], whereas the current study showed no significant difference at the RNA transcript level. Confounding factors include 1) the proportion of tumor cells present in the specimens evaluated, 2) different criteria for categorizing expression status, and 3) RNA versus protein targets.

In general, the array technology that was used in this study worked remarkably well in generating RNA profiles that were believable by virtue of distinguishing known benign *versus* malignant and gastric *versus* cervical histopathologies. Furthermore, co-expression of analytes in the same pathway or by the same infectious agent makes sense from a pathobiology and virology perspective. Interestingly, all of the cervical tissues clustered together, and benign and malignant cervical lesions were largely segregated even though the Gastrogenus v1™ test panel had not been specifically designed to achieve these endpoints. Lack of multiple co-expressed EBV mRNAs in cervical tissues reinforced what we knew about their EBV-negativity by the gold standard *EBER in situ* hybridization assay.

Among the seven genes that were significantly more expressed in gastric cancer (regardless of infection status) compared to lymphoepithelioma-like cervical cancer, four were previously reported as gastric cancer markers (*CLDN18, REG4, OLFM4, CDH17*) [55,59-63]. Two others (EPCAM epithelial cell specific trans-membrane glycoprotein, and PPARG chemokine), as well as REG4, are being explored for targeted cancer therapy [64-66]. The last of the seven, BBC3 (also called p53 up-regulated modulator of apoptosis, or PUMA) is reportedly upregulated by EBV LMP2A and reigned in by EBV miR-BART5 in cell line models [67,68], suggesting that this BCL2 family member is tightly regulated by the virus.

One of the two RNAs that was significantly higher in cervical compared to gastric cancer was *IFITM1*, which you may recall was also found to be overexpressed in infected compared to uninfected gastric cancers. Further work is needed to explore if cervical cancers (presumably human papillomavirus-infected) and EBV-infected gastric cancers share a common virus-related mechanism for overexpression of this innate immune response factor. The other gene significantly overexpressed in cervical compared with gastric cancer was *HIF1A* whose expression was associated with that of four downstream angiogenesis mediators in our panel (*VEGFA, SLC2A1, SLC2A3* and *EPAS1*) as evidenced by positive Pearson’s correlation coefficients (data not shown). If confirmed to be operative *in vivo*, HIF pathway stimulation implies that angiogenesis inhibitors are worth investigating.

Benign *versus* malignant gastric tissues tend to cluster separately on the heat map, with some exceptions. Field effect [69] or exosomal transfer of factors to adjacent regions of the local environment [70,71] could explain why some cancers and adjacent reactive tissues had similar profiles. While macrodissection was used to carefully separate benign from malignant lesions, we cannot exclude occult malignancy as a contributor to aberrant clustering.

Among the 19 genes significantly upregulated in gastric cancer compared to adjacent non-malignant gastric mucosa, most were previously reported as gastric cancer specific markers [72-76], and we now confirm that their upregulation is detectable in archival paraffin-embedded tissue. Lower levels of *GAST* (gastrin) RNA in cancer tissues could help explain the concomitant loss of the gastrin signaling factor *CHGA* (chromogranin). The most consistently downregulated factor in gastric cancer *versus* adjacent benign mucosa was the tumor suppressor gene *CDH1 (E-cadherin)* suggesting either 1) *CDH1* promoter hypermethylation [77], 2) rare germline mutation of *CDH1* associated with heritable predisposition to gastric cancer [78], or 3) downregulation of *CDH1* by EBV LMP1 as described in cell line models [79].

LMP1 was previously reported to be absent in infected gastric cancer except in rare cases [50,51,80,81]. It was therefore surprising that Nanostring nCounter array profiling showed consistent albeit low level expression of *LMP1* RNA along with virtually all of the other EBV RNAs that were tested in the infected gastric cancers. Coordinated co-expression of multiple viral genes argues that the expression is true positive. Our microarray results raise the possibility that the viral RNAs we detected are not encoding proteins or that the proteins are 1) only transiently expressed, 2) rapidly degraded, 3) localized to rare cells that are promptly recognized and destroyed by the immune system, or 4) present at such low level that traditional assays are too insensitive to detect them [82]. The nCounter test system manufacturer claims analytic sensitivity equivalent to that of rtPCR [43].

While most viral genes were expressed almost exclusively in the infected gastric cancer cohort, *EBER1* and *EBER2* were commonly expressed in each one of the benign and malignant gastric and cervical cohorts, albeit at much lower levels than was seen in each of the EBV-infected gastric cancers. Indeed, our study revealed a novel way to identify EBV-infected gastric cancer by measuring *EBER1* and/or *EBER2* RNA in archival tissue, and we have proposed thresholds that successfully distinguish infected from uninfected gastric cancer.

Support for active viral infection in infected gastric cancer patients comes from serologic evidence of higher titers against viral capsid antigen compared to EBV-negative gastric cancer patients and benign controls [83]. Low level lytic infection was previously described in mucosal lymphoid cells [31,82,84] and in infected gastric epithelial cell lines [85]. BARF1 is known to be expressed in gastric cancer where it is proposed to act as a latent rather than a lytic factor [50,51]. Using sensitive rtPCR technology, multiple EBV lytic transcripts were detected by Luo et al in gastric cancer tissues [50]. Whether active replicative infection occurs in malignant epithelial cells or in lymphoid cells remains uncertain since histochemical stains have failed to reveal a cellular source of lytic factors in gastric tissues [82].

While EBV-infected gastric cancer is biologically distinct from EBV-negative cancer in some respects, the infected counterparts still share many of the classic features previously identified as being characteristic of gastric cancer, such as specific collagens (*COL1A1, COL1A2, COL3A1), SULF1, THY1, SPP1, INHBA*, and *SPARC*[76]. These pan-gastric cancer markers might be exploited for early diagnosis or for monitoring tumor burden during therapy, especially when multiple such markers are tested in concert to maximize specificity while still capturing the heterogeneity of the disease. Biomarkers for the EBV-infected subset, such as EBV DNA and the highly expressed viral *EBER1EBER2EBNA1*, and *BRLF1* RNAs, as well as associated cellular factors confirmed in this study, represent promising targets for early detection. To the extent that any of these factors circulate in blood, they might serve as non-invasive indicators of disease analogous to what has already been achieved for two other EBV-infected neoplasms-- post transplant lymphoproliferative disorder and nasopharyngeal carcinoma. In both of these disorders, Q-PCR of circulating EBV DNA facilitates early diagnosis and in monitoring efficacy of therapy [86-88]. High levels of *EBER1* and *EBER2* RNA were measurable in plasma of 89% of nasopharyngeal carcinoma patients [89].

Antiviral therapy is becoming more accepted given its biologic underpinnings-- the viral genome is present in *every* malignant cell of a given infected cancer-- thus making the virus one of the most appealing therapeutic targets in our armamentarium. Off-the-shelf cytotoxic T cells are now available to treat selected EBV-related malignancies [90,91]. Early clinical trial data demonstrate the merits of lytic induction therapy [33,92,93]. Assessment of lytic induction by panels of tests such as the microarray system described herein could be useful for measuring the biochemical impact of an intervention and its efficacy.

Applicability of the Nanostring nCounter system to archival paraffin embedded tissue was previously reported by others [43,44], but ours is the first study to examine viral and human RNAs in concert. The test system’s ability to rapidly profile multiple RNAs generates rich data relevant to viral oncology and patient care. A major advantage is suitability for routine fixed tissue specimens including small biopsies that were previously collected, processed and stored using customary clinical methods. While microscopy is essential to assuring that representative tissue is input into the assay, the noteworthy flexibility of the test system with regard to malignant cell proportion promotes it use in clinical settings. Panels of analytes could be tailored to support different intended uses such as suitability of a subject for a specific clinical trial, or monitoring efficacy of a given regimen in serial specimens.

## Conclusions

This study demonstrates the promise of array technology to understand associations between viral and cellular factors in naturally infected gastric cancers. We showed major biologic differences between infected and uninfected cancers, between benign and malignant tissues, and between gastric and cervical cancers. While prior work indicates that the virus lies latent in malignant tissue, we found evidence of active lytic infection and virus-associated cellular changes that should be further explored. Large panels of complementary tests promote confidence in the findings and pave the way for design of practical panels to be applied in clinical trials and, once validated as useful, implemented in routine patient care.

## Materials and methods

### Patient tissue and macrodissection

Formalin-fixed, paraffin-embedded gastric adenocarcinoma tissues from the clinical archives of three hospitals in disparate parts of the world were assembled, including 30 from the University of North Carolina Hospitals in Chapel Hill, USA, 133 from Western Regional Hospital in Santa de Rosa, Honduras, and 24 from Wakayama Medical University, Wakayama, Japan. As a control, 16 paraffin embedded tissues diagnosed as lymphoepithelioma-like carcinoma of the uterine cervix were retrieved from the archives of the University of North Carolina Hospitals in Chapel Hill. All studies were done with approval of our Institutional Review Board, University of North Carolina Biomedical IRB.

On each paraffin block, nine formalin-fixed, paraffin-embedded tissue sections, each 5uM thick, were cut. One section was stained with hematoxylin and eosin so that a pathologist could mark areas containing at least 50% malignant cells among all cells present. Cancers with less tumor were still included in the study after further categorizing them as having either 1 to 25% or 25 to 50% malignant cells in marked areas of the slide. A scalpel was used to scrape and combine the marked malignant cell-rich areas from 8 unstained sections. When non-malignant mucosa from the same surgical procedure was available, the non-malignant tissue was macrodissected from unstained sections and separately prepared for expression profiling.

### Nucleic acid isolation and expression profiling

Total nucleic acid was extracted using the HighPure miR Isolation kit using the manufacturer's instructions (Roche Applied Science). Nucleic acid quality and purity were assessed by Nanodrop spectrophotometry, and a 500 ng aliquot was spiked with each of three exogenous control RNAs designed by the External RNA Controls Consortium (ERCC number 113, 147 and 163) and then frozen until RNA expression analysis on the nCounter system according to manufacturer instructions (Nanostring). Recovery of the spiked ERCC RNAs served as a control for integrity of the stored nucleic acid. Furthermore, recovery of 6 different synthetic RNAs built into the Nanostring reagent system provided confidence that that Nanostring nCounter analytic test system performed as expected.

The instrument generated a direct digital readout of the number of each RNA molecule based on hybridization of patient nucleic acid with multiplexed pairs of capture and reporter probes tailored to each RNA of interest, followed by washing away excess probes, immobilization of biotinylated capture probe-bound RNAs on a surface, and scanning color-coded bar tags on each reporter probe. A custom panel of 96 RNA assays designed for this study included 73 human mRNAs, 7 latent and 9 lytic EBV mRNA transcripts as well as *EBER1* and *EBER2* non-coding RNAs, two cytomegalovirus mRNAs, and 3 spiked ERCC RNA controls. The target human mRNAs were chosen after literature review to represent the following characteristics, 1) gastric cancer-specific analytes, 2) EBV-dysregulated factors, 3) potential pharmacogenetic biomarkers, 4) inflammatory cell markers, and 4) housekeeping controls. (See Table 1).

**Table 1:** RNAs targeted in the GastroGenus v1™ panel

**Gene symbol**	**Alternate symbol**	**Function or utility**	**Reference sequence or GeneID**
**Gastric cancer specific RNAs and gastrin signalling factors**			
REG4		Cell regeneration and growth	NM_032044.3
OLFM4		Tumor growth & cell adhesion, olfactomedin	NM_006418.3
DKK4		Embryonic development	NM_014420.2
ODAM	APin	Enamel mineralization	NM_017855
CSAG2		Drug resistance	NM_001080848.2
MIA		Growth inhibition	NM_006533.2
CYP2W1		Drug metabolism, cytochrome p450	NM_017781.2
HORMAD1		Cell cycle regulation	NM_032132.3
MMP10		Matrix metallopeptidase, remodeling	NM_002425.2
FUS		mRNA/miRNA processing	NM_004960
CLDN18		Tight junction component, claudin	NM_001002026
SERPINH1		Collagen synthesis, peptidase inhibitor, heat shock	NM_004353
THY1		Control of inflammatory cell recruitment	NM_006288
INHBA		Inhibin, inhibits hormone secretion and cell growth	NM_002192
CXCL1		Immune development and homeostasis, chemokine	NM_001511
SPARC	osteonectin	Protects from apoptosis, docetaxel response	NM_003118
SPP1		Osteogenesis, secreted phosphoprotein	NM_000582
SULF1		Cell signaling, sulfatase	NM_015170
COL1A1		Type I collagen component	NM_000088
COL1A2		Type I collagen component	NM_000089
COL3A1		Type III collagen component	NM_000090
CDH1	E-Cadherin	Cell adhesion, mutated in heritable gastric cancer	NM_004360
EPCAM		Epithelial cell adhesion	NM_002354
GAST		Stimulates stomach acid secretion	NM_000805
CDH17		Peptide transporter, gastrin signalling	NM_004063.3
CHGA		Neuroendocrine cell, gastrin signalling	NM_001275
PTGS2	COX2	Prostaglandin synthesis, gastrin signaling, druggable	NM_000963.1
MYC		Cell cycle regulator, gastrin signalling	NM_002467.3
CCND1	BCL1	cell cycle regulator, gastrin signalling	NM_053056.2
**EBV-related inflammatory response genes and NFKB signaling factors**			
PLUNC		Gastric and nasopharyngeal carcinoma	NM_130852.2
MET		Receptor tyrosine kinase, ongogene, drug target	NM_000245.2
BACH1		Transcription factor	NM_206866
BBC3	PUMA	p53 target, pro-apoptotic target of EBV mir-BART5	NM_014417
CXCL11		Leukocyte trafficking, target of EBV mir-BHRF1-3	NM_005409
CDKN1A	P21, WAF1	Cyclin-dependent kinase inhibitor, EBV miR target	NM_000389.2
FCRL3		Immune regulation, Fc receptor-like tyrosine kinase	NM_052939
CD70		T and NK cell activation, TNF ligand	NM_001252
FSCN1		Cell morphology and motility	NM_003088
TNFSF9		Antigen (Ag) processing, TNF ligand cytokine	NM_003811
BCL2L11	BIM	Activator of apoptosis, BCL2-like	NM_006538
PTEN		Tumor suppressor, EBV miR target	NM_000314.3
PCNA		DNA replication and repair, cell proliferation indicator	NM_182649.1
GPR183		G protein-coupled receptor, EBV-induced	NM_004951
MX1		Mediates antiviral response, interferon response	NM_001144925
IFITM1		Innate antiviral and interferon response	NM_003641
FCGR2B		Phagocytosis & antibody production	NM_004001
ICAM1		NFKB regulated, cell adhesion	NM_000201.2
TRAF1		NFKB regulated, TNF receptor	NM_005658.3
FCER2	CD23	NFKB-regulated B cell differentiation, IgE receptor	NM_002002.4
IL10		Anti-inflammatory cytokine regulates NFKB signalling	NM_000572.2
**Hematopoietic cell markers**			
PTPRC	CD45	Pan-hematopoietic cell marker, T & B cell signaling	NM_002838
MS4A1	CD20	B cell marker, differentiation	NM_021950
IGLL1	CD179B	B cell marker, growth	NM_020070
BANK1		B-cell marker, receptor-induced calcium mobilization	NM_017935
FAM129C		B cell marker	NM_173544
MUM1	IRF4	Late stage B cell, signaling & differentiation	NM_032853
SDC1	CD138	Plasma cell, also epithelial cell binding and signaling	NM_001006946
CD4		Helper T cells, MHC class II antigen processing	NM_000616.3
CD8A		Suppressor T cells, MHC class I antigen processing	NM_001768
CD3G	--	Pan T cell marker, intracellular signaling	NM_000073
GPR56		NK cell marker in peripheral tissues	NG_011643.1
**Pharmacogenetic factors impacting drug response**			
ERBB2	HER2	Kinase-mediated signaling, trastuzumab target	NM_004448.2
PPARG		Glucose and lipid metabolism	NM_138711.3
TYMS		Thymidylate synthase, DNA repair, 5FU response	NM_001071.2
HIF1A		Systemic response to hypoxia	NM_001530.2
EPAS1		Angiogenesis	NM_001430
VEGFA		Mitogen for endothelial cells	NM_001025366
SLC2A3	GLUT3	Glucose transporter	NM_006931.2
SLC2A1	GLUT1	Glucose transporter	NM_006516.2
**Epstein-Barr virus (EBV) RNAs:**			NC_007605.1
LMP1	BNLF1	TNF/CD40 signalling, latent phase	3783750
LMP2A		Cell survival , latent phase	3783751
EBNA1	BKRF1	Viral persistence, episome, latent phase	3783709
EBNA1,QUK		Q promoter variant, viral persistence, latent phase	3783774
EBNA2	BYRF1	Transactivator, latent phase	3783761
EBNA3A	BERF1	Immortalization, latent phase	3783762
EBNA-LP		Transactivator, latent phase	3783746
EBER1		Non-coding RNA inhibits apoptosis, latent phase	AJ507799.2 - 6629.6795
EBER2		Non-coding RNA inhibits apoptosis, latent phase	AJ507799.2 - 6956.7128
BZLF1	Zta, Zebra	Immediate early transactivator of lytic replication	3783744
BMRF1		Early lytic DNA polymerase processivity factor, TF	3783718
BHRF1		Viral BCL2 inhibits apoptosis, early lytic phase	3783706
BCRF1		Viral interleukin 10 homologue	3783689
BARF1		Soluble CSF1 receptor homologue, early lytic phase	3783772
BRLF1	Rta	Immediate early transactivator of lytic replication	3783727
BLLF1	gp350/220	Viral entry via CD21 receptor, late lytic phase	3783713
BALF5		Viral DNA polymerase, early lytic phase	3783681
BXLF1		Thymidine kinase, early lytic phase	3783741
**Human cytomegalovirus (CMV) RNAs:**			NC_006273.2
UL83	pp65	Late lytic phase	3077579
UL54	pol	CMV DNA polymerase, early lytic phase	3077501
**Housekeeper RNAs**			
CLTC		Intracellular trafficking & endocytosis	NM_004859.2
GUSB		Glucuronidase degrades glycosaminoglycans	NM_000181.1
TBP		Transcription initiation by TATA box binding protein	NM_003194.3
HPRT1		Generation of purine nucleotides	NM_000194.1

Following analysis, raw expression data was first adjusted by subtracting the mean counts of 6 negative controls in the Nanostring reagent system. (Two additional negative controls were omitted because of cross-reactivity with *EBERs*.) Negative values were adjusted to zero, and then data was normalized for 1) technical variation using the average of 6 positive controls in the Nanostring reagent system as recommended by the manufacturer, and 2) endogenous RNA amount or quality using the average of four housekeeping RNAs (*HPRT1, GUSB, CLTC* and *TBP*). To promote accurate profiling, only those 182 specimens with the highest average housekeeping RNA content were used for statistical analysis, while another 140 specimens were excluded based on low average housekeeping RNA levels. The cohort of cases for statistical analysis was comprised of 124 cancers and 58 non-malignant mucosae, while cohort of cases excluded from statistical analysis because of poor RNA quality was comprised of 80 cancers and 60 non-malignant mucosae. Heat maps were created to show median-centered expression of each gene using Cluster 3.0 and JavaTreeView software algorithms applied to log2 transformed data.

### EBV Q-PCR and *EBER* in situ hybridization

To measure viral DNA load, an aliquot of the same total nucleic acid extract that had been used for RNA profiling was subjected to quantitative PCR targeting the BamH1W segment of the EBV genome [94]. A parallel Q-PCR assay targeting the human *APOB* gene controlled for efficacy of DNA extraction was used to normalize for the number of cells represented in the PCR assay as previously described [94]. Amplification products were measured on an ABI Prism 7500 Real-Time PCR instrument using TaqMan probe and Sequence Detection System software (Applied Biosystems) [82], and results reported in copies of EBV DNA per 100,000 cells.

Viral localization to malignant cells was tested using EBV-encoded RNA *(EBER) in situ* hybridization on paraffin sections (BOND assay, Leica Microsystems) [95]. As a quality control, RNA preservation was confirmed in parallel *in situ* hybridization to poly A tails by oligo-dT probe.

### Statistics

Unsupervised hierarchical clustering of gastric cancer tissues revealed the EBV-infected and uninfected molecular classes of gastric cancer. Three additional tissue classes (cervical cancer, and benign gastrointestinal or cervical mucosae) were defined by clinicopathologic criteria. In box plots, the median and middle two quartiles are surrounded by whiskers depicting outliers which are far above or below the interquartile range (IQR) by > Q3 + 1.5*IQR or < Q1-1.5*IQR, respectively. Genes significantly differentially expressed among groups were identified using non parametric Mann-Whitney tests and the p-values were adjusted using the Bonferroni correction to account for multiple comparisons. A given RNA was classified as significantly differentially expressed if its Bonferroni adjusted p value was <0.05 and it was more differentially expressed than any single one of the four housekeeping RNAs.

## Abbreviations

EBV: Epstein-Barr virus; NU: Normalized unit.

## Competing interests

MLG is a consultant for McKesson, Abbott Laboratories, and Roche Molecular Systems and serves on the clinical advisory board of Generation Health.

## Authors' contributions

WT and HM designed and implemented experiments and analyzed data. DRM, MOM, RLD EM, KK, and NB defined clinical priorities and provided annotated case material. PFK performed statistical analysis and prepared figures. KW and OS performed histopathologic assessment of malignant cell proportions. MLG designed the assay, interpreted data, and finalized the manuscript. All Authors read and approved the final manuscript.
